# Faces capture the visuospatial attention of chimpanzees (*Pan troglodytes*): evidence from a cueing experiment

**DOI:** 10.1186/1742-9994-6-14

**Published:** 2009-07-23

**Authors:** Masaki Tomonaga, Tomoko Imura

**Affiliations:** 1 Primate Research Institute, Kyoto University, Inuyama, Aichi 484-8506, Japan; 2 Japan Society for the Promotion of Science, Kojimachi, Chiyoda, Tokyo 102-8471, Japan

## Abstract

**Background:**

Faces, as socially relevant stimuli, readily capture human visuospatial attention. Although faces also play important roles in the social lives of chimpanzees, the closest living species to humans, the way in which faces are attentionally processed remains unclear from a comparative-cognitive perspective. In the present study, three young chimpanzees (*Pan troglodytes*) were tested with a simple manual response task in which various kinds of photographs, including faces as non-informative cues, were followed by a target.

**Results:**

When the target appeared at the location that had been occupied by the face immediately before target onset, response times were significantly faster than when the target appeared at the opposite location that had been by the other object. Such an advantage was not observed when a photograph of a banana was paired with the other object. Furthermore, this attentional capture was also observed when upright human faces were presented, indicating that this effect is not limited to own-species faces. On the contrary, when the participants were tested with inverted chimpanzee faces, this effect was rather weakened, suggesting the specificity to upright faces.

**Conclusion:**

Chimpanzee's visuospatial attention was easily captured by the face stimuli. This effect was face specific and stronger for upright faces than inverted. These results are consistent with those from typically developing humans.

## Background

Faces are special stimuli for social animals, including humans. It is well known that faces are processed in a different manner from other types of complex visual stimuli. Indeed, holistic processing of the configuration of facial features is very important in face recognition, as evidenced by the face-specific inversion effect, more impaired perception, recognition and discrimination of individuals, facial expressions, etc of upside-down faces than upright faces [1]. Such unique processing of faces occurs among other nonhuman primates, especially chimpanzees [2-5], who also clearly exhibit the face-inversion effect.

Recent advances in the study of face perception in humans have clarified that faces represent special stimuli with regard to visuospatial attention as well. That is, faces capture our attention. For example, humans easily detect changes in faces during change blindness tasks, in which the observers are required to detect the changes of faces in scenes presented successively separated with a blank frame [6,7] and search very efficiently for faces embedded in a display of non-face objects [8]. Conversely, if a face is presented as one of the distractors in a search display (a singleton distractor) during a visual search for some other object (e.g., a butterfly), humans take longer to detect the target than when the face did not appear as a singleton distractor [9,10]. This phenomenon, known as stimulus-driven attentional capture [11], occurs "only when the attribute that elicits it is independent of the observer's state of attentional readiness" (p. 157). Recently, this attentional capture effect with face stimuli was observed in humans under the simpler attention-cueing task which was similar to the present task than the visual search tasks, but only when the upright faces were presented, not inverted faces [12,13].

Several aspects of chimpanzee attention are similar to those of human attention [14]. Chimpanzees have shown impaired detection of the target under the change blindness task as humans [15], and have efficiently detected faces among non-face objects [16]. Furthermore, the visuospatial attention of chimpanzees was readily captured by a task-irrelevant singleton distractor [17]. Based on these findings about face perception and attentional processing in chimpanzees, we tested whether the visuospatial attention of chimpanzees would be captured by faces presented peripherally. We hypothesized that chimpanzees would demonstrate face-driven attentional capture. To test this hypothesis, we used a simple target detection task with a "double-cueing" paradigm [12,13]. In this task, chimpanzees were required to touch the target that was presented after the cueing photographs. This task did not explicitly require the discrimination of photographs (i.e., contents of photographs were task-irrelevant), and thus would be simpler with regard to the task-demand level than the visual search task. We had already found that chimpanzees showed faster response times when the single cue appeared at the same location as the forthcoming target versus at another location [18]. Therefore, under the double-cueing paradigm, we predicted that the manual response times of the chimpanzees would be faster when the face was presented at the same location as the forthcoming target than when the target appeared at the opposite location. To verify this effect was face-specific, we also used the other type of stimuli, that is, bananas as control condition, since the food items have ecologically relevant for the chimpanzees and they readily and preferably categorize food items against the other natural objects [19].

Furthermore, to confirm the attentional capture effect was face-specific and/or generalizable to other types of faces, we additionally tested the chimpanzees after the first series of experiment using the other-species faces, that is, humans. And to test whether this effect is closely related to face-specific processing, we further tested them using inverted chimpanzee faces. Previous studies clearly showed that chimpanzees perceived human faces in very similar manner to chimpanzee faces [3-5], and exhibited deteriorated perception for inverted faces [2-5]. Thus, we predicted that the chimpanzees would show the attentional capture effect for upright human faces, but would be weakened when inverted chimpanzee faces were presented [but see [13]].

## Methods

### Participants

**Figure 1:**
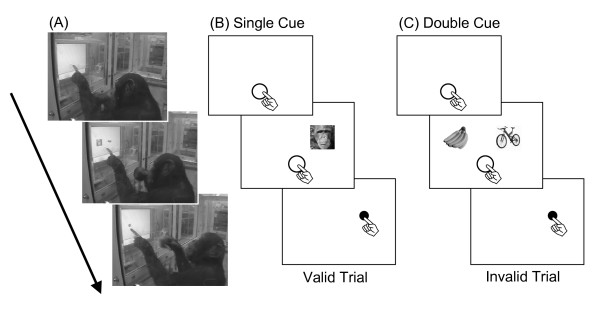
**(A) A 6-year old male chimpanzee, Ayumu, performing a valid face trial under the pair condition**. (B, C) Schematic diagram of experimental trials under single-cue and double-cue conditions. (B) and (C) also represent invalid and valid trials, respectively.

Three young chimpanzees (1 male and 2 females, aged 5–6 years during the experiments) participated in this study (Fig. 1A). They were raised by their biological mothers and are members of a social group comprised of 14 individuals living in an indoor and an environmentally-enriched outdoor compound (770 m^2^) in the Primate Research Institute, Kyoto University, Japan[20]. They have been participating in various kinds of perceptual-cognitive experiments since they were neonates [20] and learned about the computer-controlled tasks at about 1 year of age [21]. Before the present experiments, the chimpanzees had been presented with various kinds of photographs but had not experienced differential reinforcement for choosing face stimuli. No special food or water deprivation was involved in the present study. Care and use of the chimpanzees adhered to the 2002 version of the "Guide for care and use of laboratory primates" of the Institute. The research design was approved by the Animal Welfare and Animal Care Committee of the Institute.

### Materials

Experiments were conducted in an experimental booth (1.8 × 2.15 × 1.75 m) in the experimental room adjacent to the chimpanzee facility. Each chimpanzee voluntarily traveled to the booth via an overhead walkway connecting the facility and the booth. We used a 17-inch LCD monitor (1280 × 1024 pixels, pixel size: 0.264 mm × 0.264 mm) with the touch panel installed on the wall of the booth (see Fig. 1a). Viewing distance was approximately 40 cm. The food reward (small piece of apple or raisin) was delivered by a universal feeder. All equipments and experimental events were controlled by a PC.

We prepared 14 categories of color photographs (3.9 cm × 3.9 cm), including upright and inverted unfamiliar chimpanzee faces, upright unfamiliar human faces, bananas, bicycles, birds, cars, cups, flowers, houses, trains, and so on. The other categories than faces were randomly chosen by the experimenters from the photograph-file library. Each category contained five different photographs.

### Experimental Procedure

We required chimpanzees to perform a simple manual response task in which they were required to touch the target (blue dot, 1.8 cm in diameter) presented on the left or right side of the monitor as quickly as possible (Fig. 1). As mentioned below, this task did not require the participants to discriminate explicitly among stimuli, thus we did not conduct any preliminary training before testing.

Each trial began with the presentation of the warning signal (open circle, 2.4 cm in diameter) on the bottom center of the monitor after a 2-sec intertrial interval. When the chimpanzee touched the circle, a single photograph or a pair of photographs was presented for 200 msec, immediately followed by the presentation of the target at either the left or right position. When the photographs were paired, the center-to-center distance was approximately 8 cm. If the chimpanzee touched the target, the food reward was delivered with a chime sound. Note that we did not control the eye movements of the chimpanzees during the stimulus presentations, thus this task investigated the "overt" but not "covert" orienting of attention. If the chimpanzee did not respond until 6 sec passed from the onset of the target, it disappeared and the next trial began. Trials in which the chimpanzee responded faster than 150 ms or slower than 750 ms were omitted from the subsequent analyses as outliers.

Two experimental conditions were arranged for the first experiment: single- and double-cue conditions. Under the single-cue condition, only one photograph was presented before the target (Fig. 1B). If the abrupt onset of visual stimuli at the periphery captured the visuospatial attention of chimpanzees, response times would be faster when the target appeared at the same location as the photograph (valid trials) than when it was presented at the opposite side (invalid trials) irrespective of the types of photographs [18,22]. Thus, this condition was conducted to verify the current task was enough to cause attention shift by the abrupt onset of peripheral visual stimulus. In the double-cue condition, two photographs were presented horizontally (Fig. 1C). If the attention was more readily captured by the specific types of stimuli, the response time would be faster when the target appeared at the location where that stimulus was presented than vice versa.

Experimental sessions ran 5–6 days a week. Each session lasted for 10–30 minutes depending on each chimpanzee's performance. Overall, each chimpanzee received 4.7 sessions (240 ± 97 trials per session) for the single condition and 5.7 sessions (199 ± 74 trials), yielded ten 96-trial blocks under each condition. Each session contained only single- or double-cue trials and these two types of sessions were given to each chimpanzee alternately.

In 25% of the trials, an upright chimpanzee face was presented (face trials); in 25% of the trials, a banana was presented (banana trials); and in the remaining 50% of the trials, pairs of photographs in other non-face categories were randomly presented (control trials). Under the face or banana trials of the double-cue condition, photographs of a chimpanzee face and a banana were paired with photographs of the other non-face and non-banana categories. The target position was randomly assigned irrespective of the type of photographs. Trials in which the target appeared at the same location as the face or banana were designated as valid trials, and those in which the target appeared at the opposite location were designated as invalid trials. The validity of each control trial was determined randomly by a computer program. The ratio between valid and invalid trials was set to 50%, reflecting that the photographs provided no information about the position of the forthcoming target. Our hypothesis was as follows: If the visuospatial attention of chimpanzees was captured by the face stimuli, as is the case among typically developing humans, the response time would be faster when the face appeared at the same location as the forthcoming target than when it appeared at the opposite location. Furthermore, we would not find such a "validity" effect in the banana and control trials.

After completing the first series of experiment, the chimpanzees were given two additional conditions. In one condition, upright human faces were paired with other photographs in 25% of the trials (human face condition) and in the other condition, inverted chimpanzee faces were paired with other inverted photographs in 25% of trials (inversion condition). For both conditions, the rest of 75% of trials was control trials as in the previous conditions. Note that inverted photographs were presented in the control trials during the inversion condition. As in the first series of experiment, each session contained only human-face or inversion conditions, and these two types of sessions were given alternately. Each chimpanzee received 4 sessions (261 ± 102 trials per session) for human face condition and 4.3 sessions (247 ± 92 trials) for inversion condition, yielding ten 96-trial blocks for each condition. If the faster response times on valid face trials were observed in the previous face condition, and this validity effect was not due to the local features of the face stimuli but to the configural properties of faces, this effect could be generalized to other types of faces such as human faces, and would be weakened by deterioration of configural properties such as inversion (but see [13]).

### Statistical analyses

We obtained 10 blocks of data for each condition for each chimpanzee. These data were analyzed separately for each condition by general linear mixed models (using SPSS 14.0J) in which the stimulus type and validity represented fixed effects, and the participants and the blocks nested within participants served as random effects. The level of statistical significance was set at 0.05.

## Results

**Figure 2:**
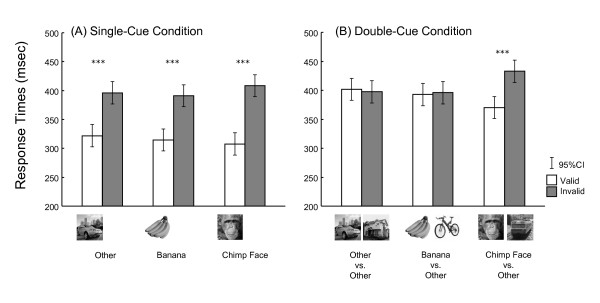
**Mean response times (in msec) for each trial type in the single-cue (A) and double-cue (B) conditions**. White bars: valid trials, gray bars: invalid trials. Error bars indicate 95% confidence intervals. ***: *p *< 0.001.

Fig. 2A shows the mean response times for single condition. As hypothesized, the chimpanzees responded significantly faster in the valid trials than in the invalid trials (315 msec vs. 399 msec) under the single-cue condition, irrespective of the type of photographs. Mixed model analysis [validity (valid, invalid) × type of trials (control, banana, chimpanzee)] confirmed these results (validity; *F*_1,145 _= 225.591, *p *< 0.001, photograph; *F*_2,145 _= 0.485, *p *= 0.617, two-way interaction; *F*_2,145 _= 2.239, *p *= 0.110). On the other hand, as shown in Fig. 2B, the validity effects varied across the type of photographs during the double-cue condition. Especially the chimpanzees responded faster when the target appeared at the same location where the chimpanzee face had been presented than the opposite side. Mixed model analysis revealed no significant main effect of type of the trials (*F*_2,145 _= 1.063, *p *= 0.348), but the significant effect of validity (*F*_1,145 _= 23.970, *p *< 0.001) and two-way interaction (*F*_2,145 _= 25.429, *p *< 0.001). Post hoc analysis clearly indicated the significant effect of the validity only for face trials (*F*_1,145 _= 74.315, *p *< 0.001), but not for control trials (*F*_1,145 _= 0.327, *p *= 0.568) and banana trials (*F*_1,145 _= 0.186, *p *= 0.667). These results are also consistent with our hypothesis.

**Figure 3:**
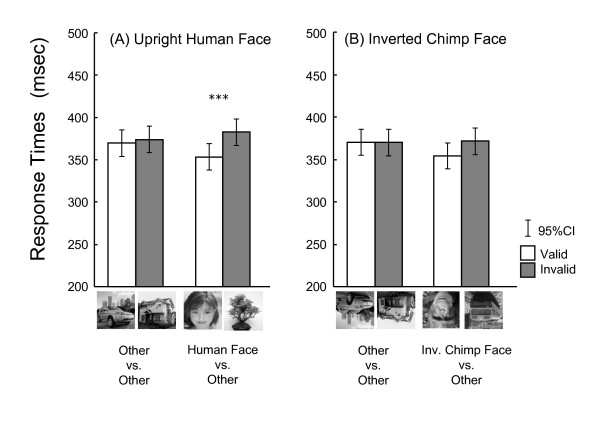
**Mean response times (in msec) for each trial type in the upright human face (A) and inverted chimpanzee face (B) conditions**. White bars: valid trials, gray bars: invalid trials. Error bars indicate 95% confidence intervals. ***: *p *< 0.001.

Fig. 3 shows the mean response times for the two additional tests. In the upright human face condition, the chimpanzees exhibited faster response times for valid face trials (353 ms) than invalid trials (383 ms, Fig. 3A). Mixed model analysis [validity (valid, invalid) × type of the trials (control, human face)] indicated no significant effect of trial types (*F*_1,87 _= 0.499, *p *= 0.482), but the significant effect of validity (*F*_1,87 _= 9.093, *p *= 0.003) and two-way interaction (*F*_1,87 _= 4.920, *p *= 0.029). Post hoc analysis indicated the significant effect of validity only for upright human face trials (*F*_1,87 _= 13.695, *p *< 0.001) but not for control trials (*F*_1,87 _= 0.318, *p *= 0.574). In the inverted chimpanzee face condition, although the chimpanzees apparently exhibited faster response times for the valid inverted face trials (354 ms) than invalid trials (371 ms), this difference did not reach to the statistical significance (trial types; *F*_1,87 _= 1.779, *p *= 0.186, validity; *F*_1,87 _= 2.447, *p *= 0.121, two-way interaction; *F*_1,87 _= 2.575, *p *= 0.112).

## Discussion

The results clearly showed that the attention of the chimpanzees shifted to the location where the face had been presented. This effect is considered specific to facial stimuli because it was generalized to human faces. Furthermore, this effect was weakened when the face was inverted, suggesting that orientation-specific spatial configurations of facial features are one of the critical factors for this effect. These results are consistent with the previous studies showing the inversion effect in face perception in chimpanzees [2-5], and recent studies using positron emission tomography (PET) and event-related potentials showing brain activity that is specific to processing faces among chimpanzees ([23] and G. Matsuda et al., personal communication).

Low-level non-facial features, such as global brightness or color hue, that were identical in all chimpanzee photographs might have caused this face-specific attentional capture. Indeed, the chimpanzee photographs (mean gray-scale value ranging from 0 (black) to 255 (white) was 117.2) were darker than the other stimuli (144.6 averaged across all the other photographs). Thus, the brightness contrast against the white background might have caused the stimulus-driven attentional capture [11]. However, this feature cannot account for the face specificity of the attention capture in the present experiments because the validity effect was also observed in response to human faces (mean gray-scale value was 155.1), which were brighter than chimpanzee faces and as bright as control photographs. Although we should examine the other local features, the conclusion thus far is that the attentional capture observed in the present experiments is specific to faces including other species.

We also found that the validity effect was weakened by the inverted presentation of faces. This fact may imply attentional capture caused by face stimuli requires configural processing of face. This speculation is consistent with our previous studies showing the superiority of upright faces in visual searches of faces performed by chimpanzees; in these, participants conducted more efficient searches for upright faces than inverted faces even when we presented human, dog, and caricatured faces as well as chimpanzee faces [3,5,16]. In some study, validity effect still remained in humans when the inverted face was presented in the similar experimental setting to ours unlike under the visual search paradigm [13,24]. Our results also exhibited the slightly positive validity effect for inverted faces (17-msec advantage for valid trials) though not significant. When comparing Figs. 2 and 3, there is a clear "practice effect" on response times during the present study (398 ms in average across all types of trials of the first 2 conditions vs. 368 ms for the latter 2 conditions). Thus, it might be possible that this practice effect masked the validity effect in the inverted face condition.

Note that, however, the inversion effect on attentional capture by faces is still controversial in humans. The discrepancy of the results from humans can be discussed on the basis of differences in task demands in some degree (detecting the non-face target among search display including a face vs. detecting the simple target cued or uncued by the facial stimuli). In humans attentional capture by the face was modulated by the to-down control [12]. Thus, it is plausible that the task demands in the present experiment might affect the top-down, voluntary components of chimpanzee's attention, resulting in the differential effect of face inversion. This speculation should be further tested in the future using various kinds of tasks.

Bindemann and Burton [13] discussed the role of eye region in the attention to the face in humans. They found no inversion effect and proposed the possibility of preserved configuration of eye region (horizontally arranged eyes) both in upright and inverted faces. This discussion is quite suggestive to interpret our results from chimpanzees. In the series of eye-tracking studies, we found different scanning patterns of faces between chimpanzees and humans [25]: both species initially fixate eye regions but the duration is of fixation to the eyes are much shorter for chimpanzees than humans. In the present study, the chimpanzees, who exhibited inversion effect for face perception but paying less attention to the eye regions where the configuration was unchanged against inversion, showed weakened effect of attentional capture by the face. These results, conversely imply that inversion effect of attentional capture by the face in humans might be weakened (or masked) by the strong attentional biases to the eye region. The role of eyes in the attention to the face is still open for investigations. This should be examined with various populations including typical and atypical developing humans [e.g. [26]] and various species of nonhuman primates with various tasks from the comparative-cognitive perspective.

## Conclusion

In this study, we examined whether faces captured the visuospatial attention of young chimpanzees as they typically capture the visuospatial attention of typically developing humans. Consistent with our hypothesis, the visuospatial attention was easily captured by the photograph of upright chimpanzee face briefly presented at periphery of visual field. This attentional capture was also observed when the upright human face was presented but was not observed when the other non-face stimuli were presented or weakened when the inverted faces were presented. These results indicated that this effect is specific to the upright face, and suggest the special role of face in the attentional processing in chimpanzees.

## Competing interests

The authors declare that they have no competing interests.

## Authors' contributions

MT conceived of the study. MT and TI designed and conducted the experiments. MT and TI analyzed and interpreted the data. MT drafted the manuscript. Both authors read and approved the final manuscript.
